# Utilization of Urinary Neopterin Levels for Pregnancy Diagnosis in Mated Giant Pandas

**DOI:** 10.3390/ani15192796

**Published:** 2025-09-25

**Authors:** He Huang, Yuliang Liu, David C. Kersey, Zongjin Ye, Rong Hou, Xianbiao Hu, Mingxi Li

**Affiliations:** 1 Chengdu Research Base of Giant Panda Breeding, The Conservation of Endangered Wildlife Key Laboratory of Sichuan Province, Chengdu 610081, China; sdliuyuliang@163.com (Y.L.); yzj1518445ars@163.com (Z.Y.); hourong@panda.org.cn (R.H.); xtt671384@163.com (X.H.); 2 Kaiser Permanente Bernard J. Tyson School of Medicine, Pasadena, CA 91101, USA; david.c.kersey@kp.org

**Keywords:** giant panda, neopterin, pregnancy, biomarker

## Abstract

Neopterin is a well-characterized marker of cell-mediated immunity. Here, we used neopterin as a biomarker to identify the cellular immunity status of breeding giant pandas throughout gestation. Urine samples were collected from captive adult female giant pandas that had entered estrus and were either naturally bred and/or artificially inseminated (AI) during the breeding seasons at the Chengdu Research Base of Giant Panda Breeding. This study was divided into two parts. In the first part, we examined the urinary neopterin and progesterone (Pg) profiles during pregnancy in parturient and nonparturient individuals. From the day of mating to the Pg peak, there was no correlation between neopterin and Pg in both parturient individuals (*n* = 3) and nonparturient individuals (*n* = 3). During the period when the Pg dropped from its peak to approximately 100 ng/mg Cr, the changes in neopterin and Pg showed a negative correlation in parturient individuals, while there was no correlation between the changes in neopterin and Pg in nonparturient individuals. Subsequently, urine samples were categorized into four phases based on the reproductive status of giant pandas: (1) Estrus phase (Es); (2) Primary Pg increase phase (S1), during which Pg levels rise from the day of mating to 100 ng/mg Cr; (3) Secondary Pg increase phase (S2), during which Pg levels rise from 100 ng/mg Cr (inclusive) to their peak value; and (4) Post-peak Pg decline phase (S3), during which Pg levels decrease from their peak (inclusive) until they drop to approximately 200 ng/mg Cr. Analysis revealed that differences between parturient and nonparturient females emerged in the S3 phase. Compared to nonparturient individuals, neopterin concentrations showed sustained elevation in parturient pandas during the S3 phase. The second part of the study, building on the findings of the first part, specifically focused on analyzing the urine samples of giant pandas during the S2 and S3 phases. Statistical analysis of an expanded sample of individuals (including those from the first part) revealed a significant difference in the neopterin mean S3/S2 ratio between the parturient group (mean S3/S2 = 1.71 ± 0.12; *n* = 14) and the nonparturient group (mean S3/S2 = 0.85 ± 0.04; *n* = 17). Correspondingly, parturient individuals gave birth 5 to 16 days after the end of the S3 phase (this time corresponds to the point when Pg levels decrease to approximately 200 ng/mg Cr). The results revealed the validity and applicability of using the neopterin mean S3/S2 ratio (1.71 ± 0.12) for diagnosing successful pregnancy in mated female giant pandas.

## Simple Summary

This novel study aimed to investigate one of the most challenging aspects of giant panda pregnancy detection. We assessed urinary neopterin, a biomarker of cell-mediated immunity, in females that did and did not become pregnant following breeding. We found a significant difference in neopterin levels between pregnant and nonpregnant females during the last several weeks of the luteal phase, after the progesterone peak. To enable the research results to have practical predictive value for pregnancy diagnosis in mated giant pandas, we detected a significant difference in the neopterin ratio, calculated as the ratio of the mean neopterin level in the post-progesterone peak phase (progesterone decreases from its peak [inclusive] to approximately 200 ng/mg Cr) to that in the pre-progesterone peak phase (progesterone levels rise from 100 ng/mg Cr [inclusive] to its peak value) between the parturient group (ratio = 1.71 ± 0.12; *n* = 14) and the nonparturient group (ratio = 0.85 ± 0.04; *n* = 17). Specifically, the neopterin ratio of the parturient group (1.71 ± 0.12; *n* = 14) proved to be a robust indicator of pregnancy status. Neopterin assessment can be performed with commercially available assays, thereby making on-site pregnancy diagnosis practical at giant panda holding facilities.

## 1. Introduction

The giant panda (*Ailuropoda melanoleuca)* is a flagship species of conservation and significant progress has improved the status of the species in the wild and in managed care. The growth of the ex situ population has been aided by an expansion and application of species-specific reproductive knowledge. Much of this knowledge comes from the definition and description of the unique reproductive biology of the female, which includes seasonal monestrous, delayed implantation, pseudopregnancy, and lactational anestrus; however most elusive has been a definitive, non-invasive pregnancy diagnostic marker [1]. This is largely due to pseudopregnancy, during which the luteal phase is indistinguishable between parturient and nonparturient females. Irrespective of gestational status, a female that completes estrus will enter a biphasic luteal phase, characterized by a primary rise in progestagens in which concentrations are consistently three to ten times greater than baseline for a variable duration (61–122 d) [2,3,4]. The ensuing secondary rise is defined by a marked and rapid rise in progestagens that peak 30 to 100 times greater than baseline over a 20- to 30-day duration and then a decline to baseline over a similar 20- to 30-day duration [2,3,4]. If the female is parturient, parturition coincides with the return of progestagens to baseline, however there are no progestagens markers of concentration, variability, or duration that differentiate parturient from nonparturient individuals. Additionally, because of these hormonal similarities, behaviors (e.g., nesting; cradling objects) and morphological changes (e.g., vulva changes; mammary growth) are also similar between parturient and nonparturient females [1].

Pregnancy diagnosis via reproductive steroids, relaxin, or glucocorticoids have proven ineffective [5,6]. Urinary ceruloplasmin demonstrated that embryonic attachment (the end of diapause) likely occurs coincident with the secondary rise progestagen peak, but its utility as a diagnostic marker is limited because the protein is relatively unstable and daily variation in concentrations requires intensive serial sampling and real-time monitoring throughout the luteal phase [7]. A study of prostaglandins also suggests that the period after the progestagen peak in the secondary rise is the critical period of conceptus development; however, the pattern and concentration of prostaglandins during the important period only subtly differ between parturient and nonparturient females [4]. Again, the period after the secondary rise progestagen peak was identified as a critical period for fetal development by subtle changes in estrogen [8]. These changes were detected via urinary estrogen indexed with urine specific gravity (USpG) to counter variations in catabolism that influence creatinine indexing. As with the above methods, the diagnostic potential of USpG-indexed estrogen is limited to the 14 days period prior to parturition [8]. Ultrasonography is widely used in captive giant panda breeding programs to detect pregnancy, and predict parturition date. However, pregnancy detection by ultrasonography is limited to the 14 days prior to birth, requires a trained and cooperating female which is often challenging during the final weeks of the cycle [9]. Given these constraints, the development of additional confirmatory non-invasive biomarkers to distinguish between pregnancy and pseudo-pregnancy is needed.

The ceruloplasmin study provided insight about the immunological changes that occur during the different phases of giant panda pregnancy and pseudopregnancy that warrant further exploration. Successful gestation in mammals requires modulation of the dam immune system to prevent the rejection of novel DNA of the developing conceptus throughout gestation, particularly during embryonic attachment and fetal development, consequently immunological markers can be used to diagnose pregnancy [10]. Neopterin, a pteridine compound, is produced by activated macrophages and dendritic cells [11] and reflects the cell-mediated immune response [12] that also correlate with reproductive cycle phases of the human female [13]. Of additional importance, neopterin is voided in the urine unchanged, and thereby serves as an important non-invasive marker of cell-mediated immunity [14]. In this study, we use neopterin as a biomarker to identify the cellular immunity status of breeding giant panda individuals throughout gestation. We aim to (i) describe the relationship between progesterone(Pg) and neopterin levels throughout the luteal phase of parturient and nonparturient giant pandas; and (ii) determine changes in neopterin secretion between parturient and nonparturient females during estrus, the primary rise in the luteal phase, and the rise and decline of Pg during the secondary rise in the luteal phase. Our ultimate goal is to determine the efficacy of noninvasive, urinary neopterin as a biomarker of pregnancy in the giant panda.

## 2. Materials and Methods

### 2.1. Animals and Urine Samples

All giant pandas included in this study were housed at the Chengdu Research Base of Giant Panda Breeding, Sichuan Province, People’s Republic of China. Urine samples were collected from captive adult female giant pandas that had entered estrus and were either naturally bred and/or artificially inseminated (AI) during the breeding seasons between 2020 and 2023. Urine collection procedures followed the method described by Kersey et al. (2010) [3]. This study was divided into two parts. In the first part, urine samples of the participating giant pandas were selected based on the preservation records of samples collected during their reproductive period, following this principle: (1) one sample every 2–3 days during the estrus period; (2) approximately one sample every seven days throughout the luteal phase. On the premise of ensuring the integrity of urine collection time and the usability of preserved samples, urine samples from 3 parturient females [Studbook (SB) 765(2023), SB966(2023), and SB1121] and 3 nonparturient females (SB561, SB801, and SB824) were ultimately screened as eligible. Subsequently, urine samples were categorized into four phases based on the reproductive status of giant pandas: (1) Estrus phase (Es); (2) Primary Pg increase phase (S1), during which Pg levels rise from the day of mating to 100 ng/mg Cr; (3) Secondary Pg increase phase (S2), during which Pg levels rise from 100 ng/mg Cr (inclusive) to their peak value; and (4) Post-peak Pg decline phase (S3), during which Pg levels decrease from their peak (inclusive) until they drop to approximately 200 ng/mg Cr. The second part of the study, building on the findings of the first part, specifically focused on analyzing giant pandas’ urine samples during the S2 and S3 phases. The urine sample selection principle was consistent with the first part, except for stage specific adjustments: approximately one sample every seven days during the S2 and S3 phases. On the premise of ensuring the integrity of urine collection time and the usability of preserved samples, a total of 24 adult female giant pandas (aged 5–18 years, including the 6 individuals from the first part) were screened as eligible subjects. Among these 24 individuals, 2 entered estrus and underwent natural breeding and/or AI three times, 3 entered estrus and underwent the same procedures twice, and the remaining 19 underwent the same procedures once. Collectively, these 24 pandas contributed 14 parturition cases and 17 nonparturition cases during the 2020–2023 breeding seasons (see Table 1 and Table 2).

### 2.2. Urinary Neopterin Analysis

Neopterin in the urine was quantified using a commercial competitive enzyme-linked immuno-sorbent assay (ELISA) kit from IBL (RE59321; IBL International GMBH, Hamburg, Germany). Prepared standard, control, and diluted urine samples (20 µL) in duplicate were added to microtiter plates. Next, 100 μL of enzyme conjugate solution, and 50 μL of neopterin antiserum solution were added to each well. After incubation (1.5h, 24 °C) in an orbital shaker (500 rpm) in the dark, unbound components were removed with a wash, and 150 µL TMB substrate solution was added to each well and allowed to incubate (10 min, 24 °C) on an orbital shaker (500 rpm). TMB stop solution (150 µL) was subsequently added. Optimal densities were determined (maximum binding = 1.00 optical density; reading filter 450 nm) on the microtiter plate reader (Multiskan MK3; Thermo Fisher Scientific, Waltham, MA, USA) within 15 min and the neopterin concentrations in the panda urine samples were determined by comparing with the standard curve. Interassay coefficients of variation (CV) for high and low controls (*n* = 18 assays) were 12.8% and 13.1%, respectively, and the intraassay CVs for high and low controls were 6.5% and 6.2%, respectively. Urine neopterin validation (Pearson correlation; *r*^2^ = 0.99, *p* < 0.05) demonstrated displacement curves parallel to those of standard hormone preparations. Recovery of added standard to urine (Linear regression; *y* = 1.0033*x* + 0.19, *r*^2^ = 0.99) demonstrated significant (*p* < 0.05) recovery.

### 2.3. Urinary Pg Metabolite and Creatinine Analyses

Pg was quantified using a previously validated and described enzyme immunoassay (CL425; C. Munro, University of California, Davis, CA, USA) [15]. Interassay coefficients of CVs for high and low controls (*n* = 24 assays) were 12.2% and 13.4%, respectively, and the intraassay CVs were <10%.

Urinary neopterin and Pg concentrations were indexed by creatinine (Cr) as previously described [15] and expressed as neopterin or Pg mass per milligram of Cr (mass/mg Cr). Urine samples with Cr < 0.1 mg/mL^−1^ were not analyzed for hormone content and were discarded.

### 2.4. Statistical Analyses

The statistical analysis was performed with SPSS (version 21.0) (Chicago, IL, USA). In the first part of the study, Pearson correlation analysis was performed to determine the relationship between urinary neopterin and Pg secretion patterns during pregnancy. The neopterin production data from the Es, S1, S2, and S3 periods were converted using the **z**-score, and then analysis of variance (ANOVA) was used to test for differences among the four reproductive states. In the second part of the study, Welch’s *t*-test was used to determine the difference in the neopterin mean S3/S2 ratio between the parturient group and the nonparturient group. The data are presented as mean ± SE; statistical differences were considered significant at *p* < 0.05.

## 3. Results

### 3.1. Changes in Urinary Neopterin Levels in Parturient Giant Pandas

**Figure 1:**
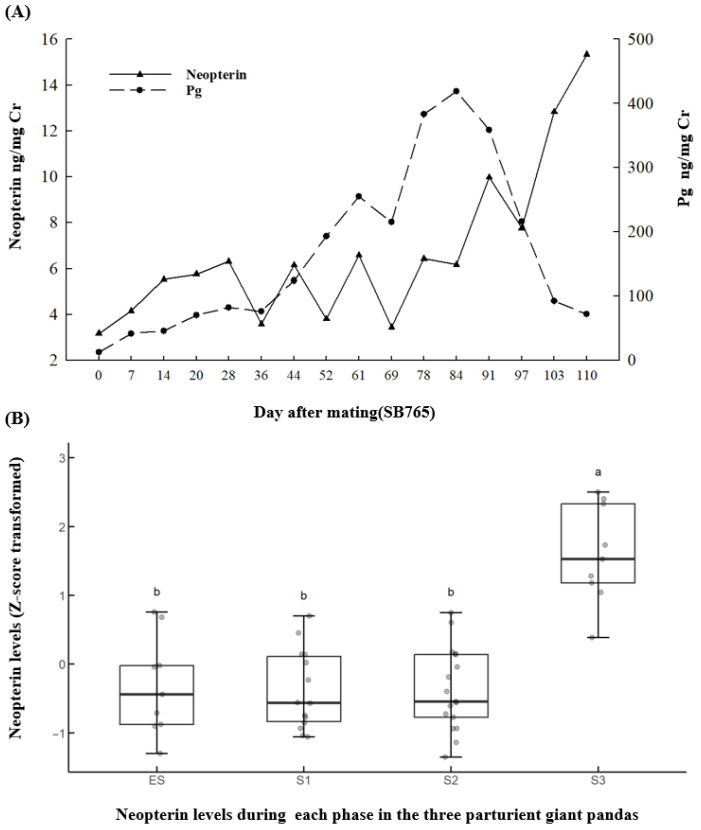
Representative pregnancy figures of: (**A**) temporal urinary neopterin and Pg profiles from the day of mating through to the period when Pg drops from its peak to approximately 100 ng/mg Cr (SB765); and (**B**) urinary neopterin levels during each phase in the three parturient giant pandas. Differences between the phases labeled with different letters were statistically significant (*p* < 0.05).

Urinary neopterin and Pg profiles from the day of mating through parturition (e.g., SB765) and urinary neopterin levels during each phase in the three parturient giant pandas SB765(2023), SB966(2023) and SB1121 are presented in Figure 1. Patterns in Pg of the giant panda (SB765) were not similar (*r* = 0.306; *p* = 0.384) to neopterin from the day of mating to the Pg peak, and Pg dropped from its peak to approximately 100 ng/mg Cr, Pg and neopterin levels showed a negative correlation(*r* = −0.941; *p* = 0.014) are presented in Figure 1A. Similar patterns in Pg and neopterin of SB966 (*r* = 0.267; *p* = 0.385) and SB1121 (*r* = 0.423; *p* = 0.262) from the day of mating to the Pg peak, and SB966(*r* = −0.967; *p* = 0.032) and SB1121 (*r* = −0.862; *p* = 0.008) during the period when Pg drops from its peak to approximately 100 ng/mg Cr.

The changes in neopterin levels at each phase were analyzed using these three parturient giant pandas. The results showed that the neopterin in S3 (10.15 ± 0.87 ng/mg Cr; *n* = 9) were significantly greater (*F*_3,45_ = 15.63, *p* = 0.001) than those in Es (5.59 ± 0.57 ng/mg Cr; *n* = 9), S1 (5.28 ± 0.39 ng/mg Cr; *n* = 14), and S2 (5.33 ± 0.46 ng/mg Cr; *n* = 17), with no differences (*p* > 0.05) among Es, S1, and S2. The data were transformed using the z-score method and plotted as shown in Figure 1B. This suggests that in the S3 phase of parturient giant pandas, the sharp decline of Pg from its peak is associated with a significant increase in neopterin levels.

### 3.2. Changes in Urinary Neopterin Levels in Nonparturient Giant Pandas

**Figure 2:**
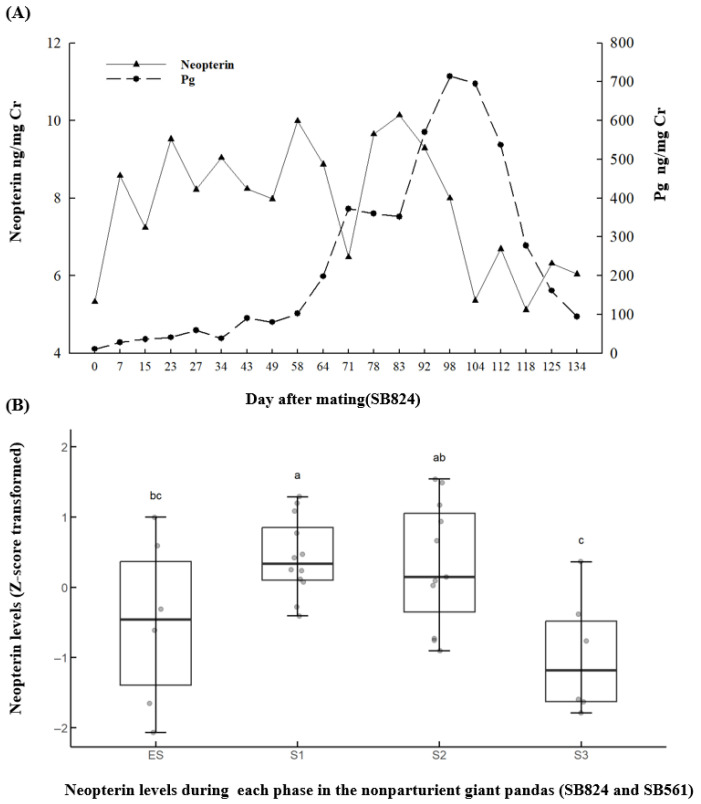
Representative pseudopregnancy figures of: (**A**) temporal urinary neopterin and Pg profiles from the day of mating through to the period when Pg drops from its peak to approximately 100 ng/mg Cr (SB824); and (**B**) urinary neopterin levels during each phase in the two nonparturient giant pandas (SB824 and SB561). Differences between the phases labeled with different letters were statistically significant (*p* < 0.05).

**Figure 3:**
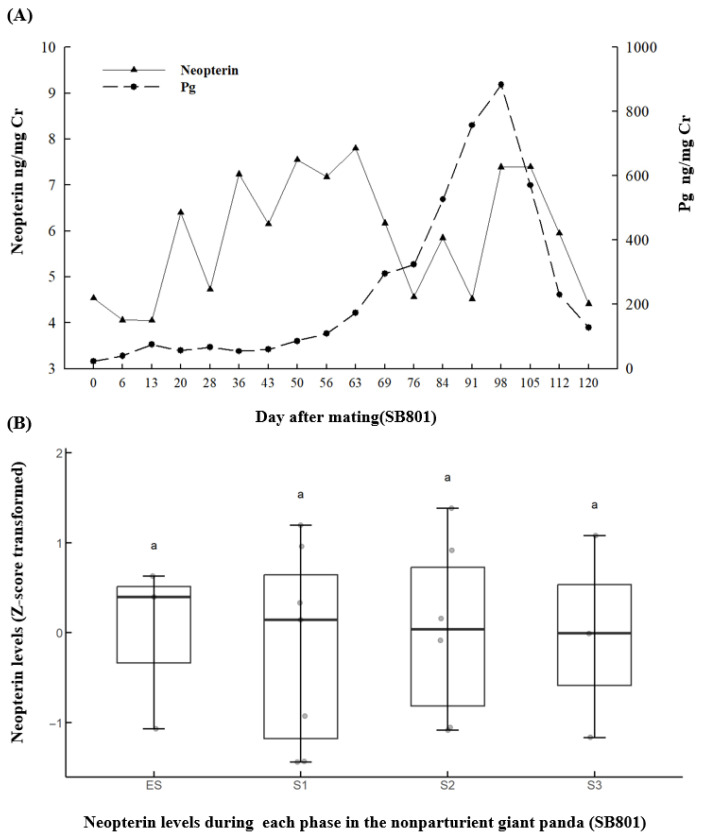
Representative pseudopregnancy figures of: (**A**) temporal urinary neopterin and Pg profiles from the day of mating through to the period when Pg drops from its peak to approximately 100 ng/mg Cr (SB801); and (**B**) urinary neopterin levels during each phase in the nonparturient giant panda SB801. Differences between the phases labeled with different letters were statistically significant (*p* < 0.05).

There are two types of patterns in Pg and neopterin of the nonparturient giant panda. Representative profiles for two types in nonparturient females are presented in Figure 2 and Figure 3. The first type is as follows: In the giant panda (SB824; Figure 2A), the pattern of Pg was not similar to that of neopterin from the day of mating to the Pg peak (*r* = 0.355; *p* = 0.234). During the period when Pg drops from its peak to approximately 100 ng/mg Cr, Pg and neopterin levels showed no correlation (*r* = 0.367; *p* = 0.456). Similar patterns were observed in giant panda SB561: the correlation between Pg and neopterin was (*r* = 0.448; *p* = 0.166) from the day of mating to the Pg peak, and (*r* = 0.685; *p* = 0.235) during the period when Pg dropped from its peak to approximately 100 ng/mg Cr. In both females, neopterin levels began to decline at or near the Pg peak and continued to decrease with fluctuations until the end of the gestation. When analyzing the neopterin levels at each phase by taking SB824 and SB561 as a whole, it was found that neopterin levels in S3 (6.19 ± 0.47 ng/mg Cr; *n* = 6) were significantly lower (*F*_3,32_ = 5.519, *p* = 0.004) than those in S1 (8.31 ± 0.23 ng/mg Cr; *n* = 12), and S2 (8.26 ± 0.40 ng/mg Cr; *n* = 12), with no differences with Es (6.88 ± 0.71 ng/mg Cr; *n* = 6). The data were transformed using the z-score method and plotted as shown in Figure 2B.

Another type is observed in SB801: From the day of mating to the Pg peak, the pattern of Pg was not similar to that of neopterin (*r* = 0.06; *p* = 0.84). During the period when Pg drops from its peak to approximately 100 ng/mg Cr, neopterin remains elevated both at the time of the Pg peak and for one week thereafter, with a decrease only beginning one week after the peak (Figure 3A). However, Pg and neopterin levels showed no correlation during this period (*r* = 0.943; *p* = 0.056). For giant panda SB801, no significant differences in neopterin levels were found among Es (5.94 ± 0.70 ng/mg Cr; *n* = 3), S1 (5.74 ± 0.55 ng/mg Cr; *n* = 7), S2 (6.01 ± 1.34 ng/mg Cr; *n* = 6), and S3 (6.28 ± 0.7 ng/mg Cr; *n* = 3) (Figure 3B; *F*_3,16_ = 0.136, *p* = 0.937). The data were transformed using the **z**-score method and plotted as shown in Figure 3B.

### 3.3. Neopterin Mean S3/S2 Ratio of Parturient vs. Nonparturient Females

To explore the differences in the patterns of neopterin changes between parturient and nonparturient females during the S2 and S3 phases, we conducted a comparative analysis of the parturient and nonparturient individuals involved in the study (Table 2). The mean S3/S2 ratio was used to illustrate the change in neopterin levels between the two phases. In parturition cases (*n* = 14), the ratio ranged from 1.03 to 2.31; in nonparturient cases (*n* = 17), it ranged from 0.56 to 1.06. The ratios were significantly greater (*t*_21.4_ = 5.8; *p* < 0.05) in the parturient group (S3/S2 = 1.71 ± 0.12) than in the nonparturient group (S3/S2 = 0.85 ± 0.04). Additionally, after the end of the S3 phase (this time corresponds to the point when Pg levels decrease to approximately 200 ng/mg Cr), parturient individuals gave birth within 5 to 16 days.

## 4. Discussion

This study provides novel evidence that pregnancy in giant pandas is accompanied by distinct immunological changes that can be detected noninvasively using a commercially available assay. Validation demonstrated that neopterin, a biomarker of cell-mediated immunity, can accurately be measured in the urine of giant panda females. Among parturient females, neopterin significantly increased during the period of pregnancy that corresponds with rapid fetal growth (S3) [9]. Additionally, nonparturient females did not demonstrate a similar increase and neopterin levels were significantly lower during S3 compared to parturient females. The identification of urinary neopterin as a reliable biomarker of cell-mediated immunity opens new avenues for understanding the maternal immune response during gestation in this species. Importantly, this approach offers a practical and scalable method for pregnancy diagnosis, addressing a long-standing challenge in giant panda reproductive management.

Successful pregnancy in mammals requires timely immunomodulation to support sperm survival, facilitate embryo implantation, and maintain fetal development. To support sperm survival and embryonic implantation in the endometrium, type 1 T helper cell (Th1) immune activity is substantially downregulated in the female reproductive tract around and after ovulation [13]. Pregnancy itself presents significant immunological challenges, as immune activity must be carefully modulated to prevent fetal loss. Although patterns may differ by cell or cytokine type, parturient females generally exhibit downregulated Th1 responses [16]. Neopterin, a marker of Th1-type cell-mediated immunity, is secreted by activated monocytes and macrophages. Elevated neopterin levels correlate with Th1-driven cellular immune activation [12]. Th1 suppression by parturient females may increase susceptibility to intracellular infection, which in turn may stimulate a cell-mediated immune response and result in higher neopterin levels [17]. Increasing neopterin levels during pregnancy are attributed to increased immunogenic stimuli by the placenta and the fetus [18]. Neopterin levels in humans, chimpanzees and bonobos with parturient and fertile females show higher levels compared with females at other reproductive stages [19,20,21], and highest neopterin levels in urine during the late pregnancy [19].

The timing of the immunological shifts coincides with key reproductive milestones, particularly the period surrounding embryo implantation and rapid fetal growth (S3 phase). This supports the hypothesis that the Pg peak marks a critical window for determining pregnancy outcome. The immunological divergence between parturient and nonparturient females bolsters the support that the period post Pg peak is the diagnostic interval for pregnancy detection in the species. During this phase, neopterin levels in parturient giant pandas increased significantly compared with those in the Es, S1, and S2 phases, whereas in nonparturient females, neopterin levels either decreased significantly or showed no difference relative to the Es, S1, and S2 phases. To enable the research results to have practical predictive value for pregnancy diagnosis in mated giant pandas. The neopterin mean S3/S2 ratio, which normalizes individual baseline variation, emerged as a robust indicator of pregnancy status. This ratio reflects the transition from pre-implantation to post-implantation immune activity and offers predictive value for determining parturition potential in mated females.

Importantly, this approach addresses a long-standing challenge in giant panda reproductive management: the inability to reliably distinguish between pseudopregnancy and true pregnancy using hormonal profiles alone. Traditional hormonal profiles, while informative about general physiological changes, have proven insufficient for reliably diagnosing pregnancy, as similar patterns are observed in both pregnant and nonpregnant females [1]. By shifting focus to immunological markers, this study reinforces previous observations that the period following the Pg peak is critical for embryo implantation and fetal development [1]. The rise in urinary neopterin during this phase suggests that successful implantation and sustained fetal growth are accompanied by measurable changes in maternal immune activity. Conversely, the absence or decline of neopterin may reflect failed implantation or early embryonic loss. These immunological patterns align with prior ultrasound-based studies, which place implantation near the Pg peak and indicate a relatively short interval between hormonal shifts and fetal detection [2,9].

There are numerous research reports on the biological functions of neopterin, and this is the first time we have applied this substance to diagnosing whether mated giant pandas will give birth. Recent studies in 2025 have reported the measurement of prolactin (PRL) levels [22]. The results showed that in female giant pandas, PRL levels increased significantly before the progestagen peak; during the luteal phase, PRL levels were significantly higher in giant pandas that gave birth than in those that did not. This study used pituitary hormones to preliminarily develop a method for diagnosing whether pregnant giant pandas would give birth. However, due to its limitation to the laboratory stage, there is still a need for practical and accessible pregnancy diagnosis methods in giant panda breeding. Currently, in practical captive giant panda breeding programs, ultrasonography is the main method for pregnancy detection, and its window for pregnancy detection is within 14 days. In this study, for the giant pandas confirmed to be parturient via the neopterin mean S3/S2 ratio, parturition occurred 5 to 16 days (*n* = 14) after the end of the S3 phase (which corresponds to Pg levels decreasing to approximately 200 ng/mg Cr). In terms of the window for pregnancy detection, the method of utilizing urinary neopterin levels for pregnancy diagnosis has not yet shown an advantage compared with ultrasonography. Notably, this non-invasive method only requires detecting neopterin and Pg in giant pandas’ urine using ELISA kits and data processing simply involves calculating the neopterin mean S3/S2 ratio. Unlike ultrasonography, it does not require a trained and cooperative giant panda, experienced testing personnel, or expensive equipment. Due to its simplicity and accessibility, both core giant panda breeding institutions and zoos around the world with small numbers of giant pandas can use this method for pregnancy diagnosis in mated giant pandas. In this way, they can make adequate preparations before the giant pandas give birth. In addition, neopterin is a small-molecule substance with no interspecific differences, so the research approach of this study can be applied to pregnancy research in other animals with embryonic diapause.

## 5. Conclusions

In conclusion, our findings demonstrate that neopterin levels differ between parturient and nonparturient mated female giant pandas during gestation, highlighting the critical role of cell-mediated immune modulation in supporting successful fetal development in parturient individuals. While the sample size in this study was relatively small, the results still validate the potential of the neopterin mean S3/S2 ratio (1.71 ± 0.12) as a biomarker for pregnancy diagnosis in giant pandas. These findings encourage us to further expand the sample size for validation and improvement, and to conduct more research on the dynamics of neopterin after pregnancy termination.

## Figures and Tables

**Table 1:** Summary of the 24 breeding female giant pandas included in this study.

Female Studbook (SB)	Mating Date *	Corresponding Parturition Date
Pregnant individuals		
SB522	17 March 2020	4 July 2020
SB537	20 May 2020	25 October 2020
SB855	6 May 2020	1 September 2020
SB870	27 March 2021	25 July 2021
SB762	8 January 2023	18 May 2023
SB966	2 February 2023	19 May 2023
SB665	8 February 2023	22 May 2023
SB635	13 February 2023	28 May 2023
SB796	9 March 2023	5 July 2023
SB765	20 March 2023	15 July 2023
SB990	27 March 2023	19 July 2023
SB870	1 April 2023	21 July 2023
SB1121	2 April 2023	22 July 2023
SB853	6 April 2023	25 August 2023
Nonparturient individuals		
SB637	22 March 2021	
SB523	7 March 2020	
SB870	10 March 2020	
SB680	19 March 2020	
SB765	23 March 2020	
SB593	24 March 2020	
SB635	21 February 2021	
SB796	23 March 2021	
SB796	27 March 2022	
SB966	6 May 2022	
SB965	9 January 2023	
SB824	20 February 2023	
SB997	23 February 2023	
SB561	27 February 2023	
SB763	2 April 2023	
SB681	26 April 2023	
SB801	26 April 2023	

Note: * All females on the mating date were naturally or artificially bred.

**Table 2:** Neopterin profiles in S2 and S3 of parturient vs. nonparturient females.

Parturient Individuals	Neopterin Level in S2 $(\bar{x}$ ±s E, ng/mg Cr)	Neopterin Level in S3 $(\bar{x}$ ±s E, ng/mg Cr)	Ratio (Neopterin Mean S3/S2)	Days *
SB870	9.01 ± 1.97 (*n* = 8)	20.80 ± 1.72 (*n* = 3)	2.31	6
SB990	6.41 ± 0.87 (*n* = 6)	14.45 ± 2.63 (*n* = 4)	2.25	5
SB966	3.44 ± 0.93 (*n* = 6)	7.32 ± 0.53 (*n* = 3)	2.13	12
SB870	14.31 ± 3.28 (*n* = 6)	30.18 ± 5.68 (*n* = 3)	2.11	9
SB665	6.65 ± 1.15 (*n* = 6)	13.45 ± 2.42 (*n* = 4)	2.02	9
SB1121	6.46 ± 0.96 (*n* = 6)	12.9 ± 1.08 (*n* = 3)	2.00	7
SB796	5.42 ± 0.62 (*n* = 6)	10.00 ± 1.97 (*n* = 3)	1.85	10
SB765	4.89 ± 0.93 (*n* = 5)	7.97 ± 1.10 (*n* = 3)	1.63	16
SB635	7.72 ± 0.72 (*n* = 5)	12.15 ± 1.80 (*n* = 4)	1.57	11
SB853	5.52 ± 0.58 (*n* = 7)	7.62 ± 0.76 (*n* = 4)	1.38	7
SB522	7.74 ± 0.69 (*n* = 6)	10.37 ± 0.85 (*n* = 3)	1.34	9
SB537	10.62 ± 1.05 (*n* = 6)	12.79 ± 2.54 (*n* = 3)	1.20	14
SB762	13.6 ± 2.16 (*n* = 5)	15.66 ± 2.17 (*n* = 4)	1.15	10
SB855	11.36 ± 0.46 (*n* = 8)	11.72 ± 0.12 (*n* = 4)	1.03	14
Nonparturient individuals				
SB681	4.59 ± 0.77 (*n* = 5)	4.88 ± 0.39 (*n* = 4)	1.06	
SB763	12.15 ± 1.63 (*n* = 7)	12.93 ± 1.62 (*n* = 4)	1.06	
SB523	3.95 ± 0.59 (*n* = 8)	4.17 ± 0.47 (*n* = 4)	1.05	
SB801	6.01 ± 0.54 (*n* = 6)	6.28 ± 0.71 (*n* = 3)	1.04	
SB765	10.60 ± 1.41 (*n* = 6)	10.71 ± 1.53 (*n* = 3)	1.01	
SB997	11.15 ± 0.70 (*n* = 6)	11.16 ± 1.19 (*n* = 4)	1.00	
SB966	8.56 ± 0.86 (*n* = 5)	8.51 ± 1.37 (*n* = 3)	0.99	
SB965	7.87 ± 0.91 (*n* = 4)	7.33 ± 0.88 (*n* = 4)	0.93	
SB796	12.87 ± 3.32 (*n* = 7)	10.96 ± 3.3 (*n* = 3)	0.85	
SB561	22.79 ± 0.97 (*n* = 5)	16.46 ± 1.36 (*n* = 3)	0.85	
SB637	9.46 ± 0.65 (*n* = 8)	7.11 ± 1.82 (*n* = 4)	0.75	
SB593	8.62 ± 2.31 (*n* = 6)	6.19 ± 0.54 (*n* = 4)	0.72	
SB635	17.09 ± 5.16 (*n* = 5)	12.12 ± 1.08 (*n* = 3)	0.71	
SB796	19.13 ± 0.72 (*n* = 6)	12.96 ± 0.16 (*n* = 3)	0.68	
SB824	8.74 ± 0.54 (*n* = 6)	5.72 ± 0.49 (*n* = 3)	0.65	
SB870	12.75 ± 2.91 (*n* = 5)	7.86 ± 1.45 (*n* = 3)	0.62	
SB680	10.00 ± 1.30 (*n* = 6)	5.58 ± 0.50 (*n* = 4)	0.56	

Note: * After the end of the S3 phase (this time corresponds to the point when Pg levels decrease to approximately 200 ng/mg Cr), parturient individuals gave birth within 5 to 16 days.

## Data Availability

The data that support the findings of this study are available from the corresponding author upon reasonable request.
